# The “χ” of the Matter: Testing the Relationship between Paleoenvironments and Three Theropod Clades

**DOI:** 10.1371/journal.pone.0147031

**Published:** 2016-02-01

**Authors:** Marcos A. F. Sales, Marcel B. Lacerda, Bruno L. D. Horn, Isabel A. P. de Oliveira, Cesar L. Schultz

**Affiliations:** 1 Departamento de Paleontologia e Estratigrafia, Instituto de Geociências, Universidade Federal do Rio Grande do Sul (UFRGS), Porto Alegre, Rio Grande do Sul, Brazil; 2 Serviço Geológico do Brasil (CPRM), Superintendência Regional de Recife, Pernambuco, Brazil; 3 Instituto Nacional de Pesquisas da Amazônia (INPA), Manaus, Amazonas, Brazil; Museum für Naturkunde, GERMANY

## Abstract

The view of spinosaurs as dinosaurs of semi-aquatic habits and strongly associated with marginal and coastal habitats are deeply rooted in both scientific and popular knowledge, but it was never statistically tested. Inspired by a previous analysis of other dinosaur clades and major paleoenvironmental categories, here we present our own statistical evaluation of the association between coastal and terrestrial paleoenvironments and spinosaurids, along with other two theropod taxa: abelisaurids and carcharodontosaurids. We also included a taphonomic perspective and classified the occurrences in categories related to potential biases in order to better address our interpretations. Our main results can be summarized as follows: 1) the taxon with the largest amount of statistical evidence showing it positively associated to coastal paleoenvironments is Spinosauridae; 2) abelisaurids and carcharodontosaurids had more statistical evidence showing them positively associated with terrestrial paleoenvironments; 3) it is likely that spinosaurids also occupied spatially inland areas in a way somehow comparable at least to carcharodontosaurids; 4) abelisaurids may have been more common than the other two taxa in inland habitats.

## Introduction

Paleontology, as a science which deals with ancient life, was never solely a descriptive activity; therefore, some attempts to “resurrect” extinct organisms can be found since its earliest days. This task must rely on empirical evidence, comparisons with modern analogues, and biomechanical modeling [1], along with methodological frameworks, like the Extant Phylogenetic Bracket [2], and new technologies, like computed tomography and isotopic analyses [3–8]. In this regard, dinosaurs are common targets of these approaches and they are undeniably good examples of the turnovers of ideas and (mis)conceptions about the way the ancient ecosystems have been seen [9].

Solving the puzzle of dinosaur paleoecology also requires investigations on their spatial niche, which are relatively few in number when compared with, for example, works focusing on diet and feeding habits, even though some studies focusing on the latter may also encompass the former [5, 10, 11]. In general, this kind of inference is scattered among anatomical and morphological statements and arises from more restricted or qualitative assessments of the patterns of the fossil record [9, 11–15]. Butler and Barrett [15] designed a simple but logical way of testing the relationships between paleoenvironments and Cretaceous herbivorous dinosaur taxa. They first collected occurrence data of all these taxa and classified them by their respective paleoenvironments, i.e., terrestrial, coastal, and marine. Then, the Chi-square tests were used to identify associations between clades and paleoenvironments. Thus, they were able to corroborate previous hypotheses of overrepresentation of nodosaurid ankylosaurs and hadrosaurid ornithopods in marine sediments [12, 13], whilst they also found that marginocephalians, ankylosaurid ankylosaurs, sauropods, and supposedly herbivorous theropods were positively associated with terrestrial paleoenvironments.

Spinosaurid theropods are another dinosaur taxon for which there are hypotheses linking them to particular habitats. Their crocodile-like skulls along with evidence from gut contents, histological thin sections and isotopic data seem to corroborate an inferred semi-aquatic lifestyle (e.g., [7, 16–21]) and, for some, it may also indicate or be related to a possible preference for marginal and coastal habitats [19, 21]. This sort of conception is not present only in scientific literature, but also in the popular view regarding these animals, fed by documentary shows, some of which also proposing a linkage between the extinction of these animals and the loss of their habitats due to the rise of sea levels during the beginning of the Late Cretaceous [22].

If there is a positive association between spinosaurs and any sort of paleoenvironment in comparison with other theropod taxa, the approach mentioned above is supposed to be able to identify it. Thus, here we present the results of such a test. In addition to spinosaurid theropods, we included in this analysis two other taxa, Abelisauridae and Carcharodontosauridae. We chose them because: 1) they are theropod taxa generally regarded as inhabiting terrestrial settings; 2) they are the less inclusive medium to large-bodied theropod clades with some specimens found in the same formations of spinosaurs, which *may* indicate some level of sympatry among them (e.g., [20, 23–26]); and 3) they are related to the episode of faunal turnover in Gondwana during the Late Cretaceous, mirrored by the one in North America: spinosaurids and carcharodontosaurids went extinct, whilst abelisaurids became the top predators [27–30]. Dealing with a smaller number of occurrences than Butler and Barrett [15] also enabled us to take into account the effects of other variables as explained below.

## Material and Methods

### Compilation and classification of occurrences

The worldwide occurrences of Abelisauridae, Carcharodontosauridae, and Spinosauridae were compiled consulting the Paleobiology Database (PaleoDB) through the Fossilworks webpage (see **S1 Appendix** for further details) as the primary source of such data. The occurrences listed therein were later compared to the literature in order to evaluate their validity (e.g., [31]). Hence, we were able to both remove and add occurrences to the PaleoDB list for this analysis. The removal of an occurrence was performed when the references listed by the PaleoDB in fact did not indicate the presence of a particular taxon, neither did any additional reference. We also removed from our analysis those occurrences based on footprints, as they are not diagnostic for any large-bodied theropod clade of our interest. On the other hand, we added occurrences when we found references that were not present in the PaleoDB. This was especially the case of recent papers, so we took into account references published until December 31, 2014. As some occurrences of the PaleoDB were based also on conference abstracts and other scientific meeting papers, we also included this kind of reference when they were not listed by the PaleoDB. However, all occurrences based on this type of publications were considered as dubious (see below).

**Fig 1:**
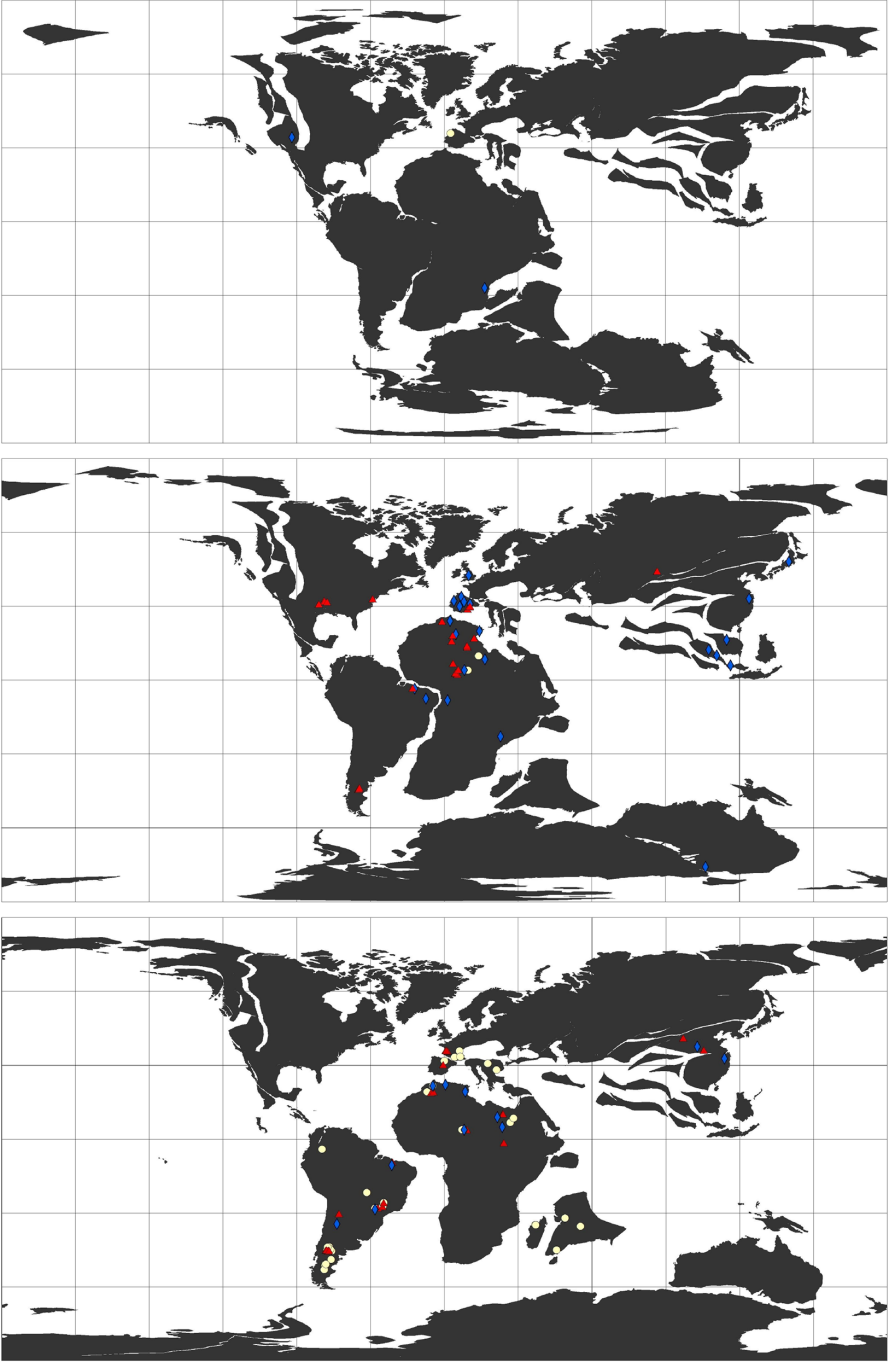
Global occurrences of Abelisauridae (white circles), Carcharodontosauridae (red lozenges), and Spinosauridae (blue triangles). From top to bottom: Late Jurassic, Early Cretaceous, and Late Cretaceous paleomaps. For the paleogeographic reconstructions and plotting the occurrences it was used the software Point Tracker [32].

We compiled a total of 198 localities (Fig 1; **S1 Dataset**), some of which representing the occurrence of only one of the taxa mentioned above, while others were shared by two or all three families. Spinosauridae had the greatest number of occurrences (82), followed by Abelisauridae (72), and then Carcharodontosauridae (66). These occurrences were then classified according to the following broad paleoenvironmental categories proposed by Butler and Barrett [15]: terrestrial (166), coastal (25), and marine (7). In order to do this, the localities were checked for stratigraphic, sedimentologic, and paleoenvironmental studies. Although the PaleoDB represented the main basis of our dataset, our classification regarding the paleoenvironments differed partially (see details ahead) from that of the PaleoDB and also from that of Butler and Barrett [15] for those localities listed by them and shared with our study.

Although Butler and Barrett [15] mentioned that some of their results were not easily explained by taphonomy or by selective transportation, they did not use any taphonomic parameter other than the body plan of the analyzed taxa and this was performed only as a qualitative assessment. However, as already historically observed by Sternberg [12], preferences for particular habitats should have had implications over the fossil record, with those taxa which inhabited closer to the depositional environment having a more complete and better preserved fossil record than that of taxa which inhabited further [33–35]. So, in order to take this issue into account as far as it was possible regarding our dataset, we also divided the occurrences in two broad taphonomic categories: category 1, formed by those occurrences based on records *including* cranial and/or postcranial remains found (semi)articulated or associated; and category 2, corresponding *solely* to those occurrences with records of isolated and fragmentary materials (see **S1 Dataset**). It is the assumption of the present analysis that these broad categories are more related to transportation than to other factors like anatomical peculiarities, especially where two or more taxa were found together and present different fossil records with respect to completeness, because abelisaurids, carcharodontosaurids, and spinosaurids have generally similar body plans and overlap in size. However, some references just pointed the occurrence of a taxon in a given locality without detailing its fossil record. Those cases were considered as dubious (see below) and also excluded specifically from analyses of broad taphonomic categories.

### Problematic occurrences

Now, it may be appropriate to specify the concept of “occurrence”. Butler and Barrett [15] defined it as “the presence of a particular taxon at a particular locality”. In this work, an occurrence is the presence of a particular taxon at a particular locality and *time*. Although, in most cases, Butler and Barrett’s [15] concept of occurrence is, in practice, also specific regarding time, there are occasions in which it is not true. For instance, this is the case of the occurrence of Spinosauridae in the Late Jurassic of Tanzania. For the PaleoDB the presence of two isolated teeth attributable to spinosaurids is counted as a single occurrence. However, as Buffetaut [36] indicated that they come from different stratigraphic levels with distinct ages, we consider each tooth as a single occurrence, so in our dataset there are two occurrences of spinosaurids in Tanzania (**S1 Dataset**). We adopted the same procedure whenever possible.

**Fig 2:**
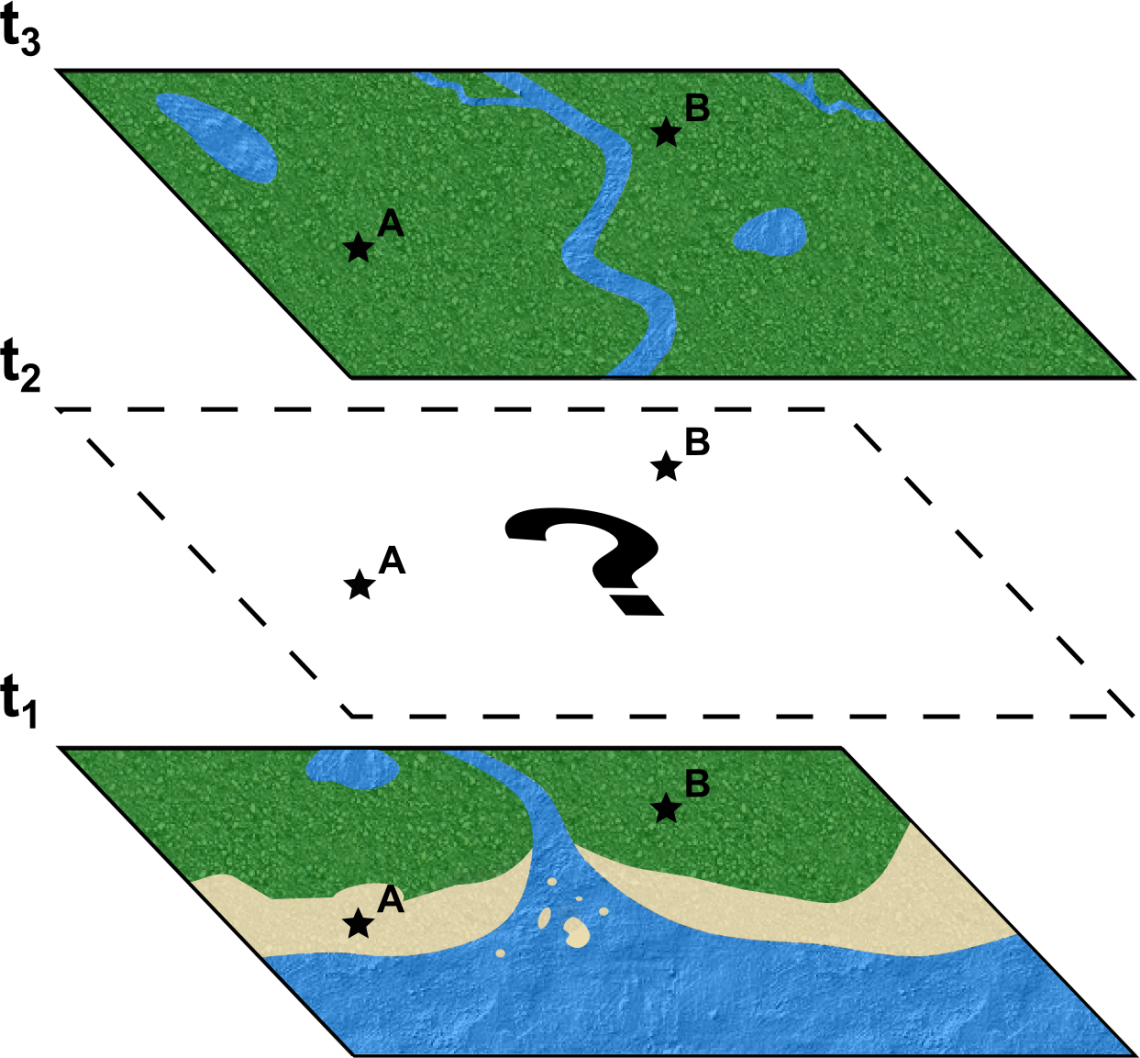
Schematic illustration of the concept of possibly paralogous occurrences. Consider two distinct localities A and B indicated by dark stars. In a given time t1, A and B are placed in distinct paleoenvironments, coastal and terrestrial, respectively. However, in t3, A and B are part of the same broad ecosystem, so counting these localities as distinct occurrences leads to the overrepresentation of a particular fossil taxon, present in both localities, in this paleoenvironment within the dataset. Thus, distinct localities and occurrences pertaining to the same stratigraphic units and ages and classified as the same broad paleoenvironment are considered as possibly paralogous occurrences. Also, locality B is part of terrestrial paleoecosystems in both t1 and t3, so those paleoecosystems may be the same throughout the time span between t1 and t3. However, as usual, the sedimentary and, consequently, the fossil records may be fragmentary and doubtful (t2), so it is not possible to track the entire paleoenvironmental history of locality B and, hence, be sure if it represents the same paleoenvironment in t1 and t3.

This leads to other questions. For instance, what about two localities that belonged to the same paleoenvironment? If they are counted individually we may be overestimating the presence of a particular taxon in a particular environment (Fig 2). We called these occurrences as “possibly paralogous occurrences”. Although possibly paralogous occurrences encompass mainly localities which pertain to the same geological formation and are close to each other, clearly it will not be the case for close localities classified as different paleoenvironments, i.e., terrestrial and coastal, terrestrial and marine, or coastal and marine (Fig 2). For practical purposes, we considered all occurrences pertaining to the same stratigraphic unit and age that are attributable to the same broad paleoenvironmental category as possibly paralogous occurrences (**S1 Dataset**). Distinct fossils coming from close localities, but lacking detailed stratigraphic data were also considered as possibly paralogous occurrences.

Questions may arise regarding the possibility of occurrences also being paralogous in relation to time. This possibility is real because one paleoenvironment might have existed for a time long enough to be represented in different stratigraphic levels. However, keeping in mind that the sedimentation is rather episodic and that there are many gaps in the stratigraphic sequence, it may be argued that it is not possible to rule out the hypothesis of these same paleoenvironments being temporally unrelated and distinct from each other (Fig 2). Due to the virtual impossibility of evaluating all of these parameters and that many sedimentary deposits lack a detailed stratigraphic analysis, we limited the concept of paralogy to the criteria mentioned in the previous paragraph.

Another issue that pervades this kind of analysis is the taxonomic one: different authors, different taxonomic attributions. We followed recent taxonomic reviews and phylogenies for our taxonomic assignments (e.g., [37–39]). However, different assignments are sometimes symptomatic of the fragmentary nature of the fossil record. Furthermore, some occurrences listed in the PaleoDB are based on references that did not figure the material attributed to a particular taxon. This was the case of some complete papers and abstracts published in some annals (e.g., [40–44]). These occurrences were considered as dubious. Again, for practical purposes, those occurrences based on a single tooth, which correspond to a relevant portion of our dataset, were also kept as dubious (e.g., [45–48]). One special case is that of the post-Cenomanian Brazilian occurrences of carcharodontosaurids. Due to their questioned identities because of temporal unconformity with other global occurrences, they were also considered as dubious. All dubious occurrences are indicated in **S1 Dataset**.

There are practical implications when considering some occurrences as possibly paralogous and/or dubious. As detailed below, we performed statistical tests including and excluding those kinds of problematic occurrences. So, when two or more occurrences were considered as possibly paralogous, they were counted only once for the tests excluding paralogy, a procedure we called as “synonymization of occurrences”. When the paralogy was between valid and dubious occurrences, the occurrence resulted from the synonymization procedure was no longer considered as dubious. Also, when the paralogy was between those pertaining to different taphonomic categories, the combined occurrences were included in category 1 after being synonymized. In short, the number of occurrences analyzed by the tests excluding both paralogous and dubious ones was not simply their total number minus the number of both possible paralogies and dubious records, especially when considering the taphonomic categories (see below).

### The statistical tests

Butler and Barrett [15] used the Chi-square tests to identify significant associations between herbivorous dinosaur clades and each type of paleoenvironment. As stated by them, a positive or negative association obtained for a given taxon does not have to do with it being only or never found in that environment, respectively. Actually, it means that this taxon has a greater or fewer number of occurrences in a certain environment than expected if all taxa are assumed to be distributed evenly across all environments. On the other hand, the absence of a significant association between a taxon and a paleoenvironment suggests that the number of its occurrences is within the range predicted by probability models.

The statistical treatment of each paleoenvironment separately deserves some consideration. This sort of test is based on a table of several lines (according to the number of taxa) and one column (a particular paleoenvironment). Thus, in practice, this means that the way the expected value for the occurrences of each taxon in this particular environment is calculated is a simple division of all occurrences in that paleoenvironment by the number of taxa. So, this approach does not exactly take into account the fact of some taxa being more widespread distributed than others. The same reasoning is applicable for the tests we performed here regarding the taphonomic categories, as there is no logical basis for inferring the same expected value of occurrences for each category within each paleoenvironment–clearly, the number of occurrences based on isolated materials is greater than that of more complete records as it is expected.

As our study was partially based on Butler and Barrett’s [15] approach, we performed the same tests with Abelisauridae, Carcharodontosauridae, and Spinosauridae, including analysis of the taphonomic categories, using the software R version 3.1.1 [49]. After the compilation of all occurrences, we excluded those in marine environments due to their very low number (Tables 1, 2 and 4). However, in order to reduce the problems cited in the last paragraph regarding the expected values of different taxa and our broad taphonomic categories, we used two additional approaches. The first of them was performing tests with more than one environment at the same time (Tables 3 and 5), because adding a new column, i.e., a new paleoenvironment, changed the way the expected value was calculated. In contingency tables with more than one line and more than one column, the expected value for each cell is calculated in terms of probability–the expected value of each cell is the chance of a sample pertaining to the same line of the cell multiplied by the chance of a sample pertaining to the same column of the cell and the total number of samples or observations [50]. The second approach was testing the relationship between taphonomic categories and paleoenvironments for each taxon separately (Tables 6–8). In this case in particular, our contingency tables were 2x2 (two taphonomic x two paleoenvironmental categories), so we used Fisher’s exact test in addition to the Chi-square tests as a supplementary source of corroboration (or refutation) of the results obtained. As most taxa of this study are Cretaceous in age, we also verified the relation between paleoenvironmental categories and epochs, i.e., Early Cretaceous and Late Cretaceous (Table 9). We excluded the Jurassic occurrences only from this analysis in particular for the same reasons for the exclusion of marine occurrences–the very low number.

**Table 1:** Different datasets of occurrences.

Taxon or Period	Taphonomic Category or Epoch	Dataset I	Dataset II	Dataset III	Dataset IV				
		C	T	C	T	C	T	C	T
**Abeli. (72)**	**1**	1	26	1	12	1	24	1	11
	**2**	2	37	2	13	1	29	1	8
	**Not specified**	0	0	0	0	0	0	0	0
**Carch. (66)**	**1**	0	15	0	11	0	15	0	10
	**2**	9	41	6	20	5	27	5	13
	**Not specified**	1	0	1	0	0	0	0	0
**Spino. (82)**	**1**	6	12	3	10	5	10	3	8
	**2**	8	52	7	20	4	32	4	13
	**Not specified**	2	1	1	1	0	0	0	0
**Cretaceous (194)**	**Early**	14	77	9	34	10	54	8	27
	**Late**	8	88	4	36	3	74	3	28

C and T refer to the costal and terrestrial paleoenvironmental categories, respectively, while taxa are indicated by Abeli. (Abelisauridae), Carch. (Carcharodontosauridae), and Spino. (Spinosauridae). On the other hand, taphonomic categories are indicated by their respective numbers except for those occurrences lacking data about the nature of their fossil record, whose taphonomic categories were considered as not specified. Within brackets is the total number of occurrences of each taxon or period.

**Table 2:** Results of Chi-square Test 1 as presented by software R.

Test 1	Dataset I	Dataset II	Dataset III	Dataset IV				
	C	T	C	T	C	T	C	T
**Abeli.**	-	n/a	n/a	n/a	n/a	n/a	n/a	n/a
**Carch.**	+	n/a	n/a	n/a	n/a	n/a	n/a	n/a
**Spino.**	+	n/a	n/a	n/a	n/a	n/a	n/a	n/a
**χ**^**2**^	8.7586	0.7283	4.5714	0.8276	4.625	1.7664	2.7143	0.381
**p-value**	0.01253*	0.6948	0.1017	0.6611	0.09901	0.4135	0.2894^‡^	0.8266

C and T refer to the coastal and terrestrial paleoenvironmental categories, respectively, while taxa are indicated by Abeli. (Abelisauridae), Carch. (Carcharodontosauridae), and Spino. (Spinosauridae). Positive, negative or lack of any association are signalized by +, -, and n/a, respectively. For the residual analysis values that indicate the type of association see **S1 File**.

* Significant p-value.

^‡^ p-value obtained in Chi-square test using the Monte Carlo analysis.

**Table 3:** Results of Chi-square Test 2 as presented by software R.

Test 2	Dataset I	Dataset II	Dataset III	Dataset IV				
	C	T	C	T	C	T	C	T
**Abeli.**	-	+	n/a	n/a	n/a	n/a	n/a	n/a
**Carch.**	+	-	n/a	n/a	n/a	n/a	n/a	n/a
**Spino.**	+	-	n/a	n/a	n/a	n/a	n/a	n/a
**χ**^**2**^	7.3431	2.6081	5.5498	1.9352				
**p-value**	0.02544*	0.2714	0.07246^‡^	0.4308^‡^				

C and T refer to the coastal and terrestrial paleoenvironmental categories, respectively, while taxa are indicated by Abeli. (Abelisauridae), Carch. (Carcharodontosauridae), and Spino. (Spinosauridae). Positive, negative or lack of any association are signalized by +, -, and n/a, respectively. For the residual analysis values that indicate the type of association see **S1 File**.

* Significant p-value.

^‡^ p-value obtained in Chi-square test using the Monte Carlo analysis.

**Table 4:** Results of Chi-square Test 3 as presented by software R.

Test 3	Dataset I	Dataset II	Dataset III	Dataset IV				
	C	T	C	T	C	T	C	T
**Abeli. 1**	-	-	-	n/a	n/a	+	n/a	n/a
**Abeli. 2**	-	+	-	n/a	n/a	+	n/a	n/a
**Carch. 1**	-	-	-	n/a	n/a	-	n/a	n/a
**Carch. 2**	+	+	+	n/a	n/a	+	n/a	n/a
**Spino. 1**	+	-	-	n/a	n/a	-	n/a	n/a
**Spino. 2**	+	+	+	n/a	n/a	+	n/a	n/a
**χ**^**2**^	16.9231	39.918	12.2632	7.0698	9.5	16.0657	8.2857	2.4286
**p-value**	0.0065*^‡^	1.55E-07*	0.03698*^‡^	0.2155	0.09845^‡^	0.006659*	0.1554^‡^	0.7872

C and T refer to the coastal and terrestrial paleoenvironmental categories, respectively, while taxa are indicated by Abeli. (Abelisauridae), Carch. (Carcharodontosauridae), and Spino. (Spinosauridae). Numbers after the taxa represent the taphonomic categories. Positive, negative or lack of any association are signalized by +, -, and n/a, respectively. For the residual analysis values that indicate the type of association see **S1 File**.

* Significant p-value.

^‡^ p-value obtained in Chi-square test using the Monte Carlo analysis.

**Table 5:** Results of Chi-square Test 4 as presented by software R.

Test 4	Dataset I	Dataset II	Dataset III	Dataset IV				
	C	T	C	T	C	T	C	T
**Abeli. 1**	-	+	n/a	n/a	-	+	n/a	n/a
**Abeli. 2**	-	+	n/a	n/a	-	+	n/a	n/a
**Carch. 1**	-	+	n/a	n/a	-	+	n/a	n/a
**Carch. 2**	+	-	n/a	n/a	+	-	n/a	n/a
**Spino. 1**	+	-	n/a	n/a	+	-	n/a	n/a
**Spino. 2**	+	-	n/a	n/a	+	-	n/a	n/a
**χ**^**2**^	14.6137	5.3791	13.8029	5.3592				
**p-value**	0.01249*^‡^	0.3923^‡^	0.01549*^‡^	0.3758^‡^				

C and T refer to the coastal and terrestrial paleoenvironmental categories, respectively, while taxa are indicated by Abeli. (Abelisauridae), Carch. (Carcharodontosauridae), and Spino. (Spinosauridae). Numbers after the taxa represent the taphonomic categories. Positive, negative or lack of any association are signalized by +, -, and n/a, respectively. For the residual analysis values that indicate the type of association see **S1 File**.

* Significant p-value.

^‡^ p-value obtained in Chi-square test using the Monte Carlo analysis.

**Table 6:** Results of Chi-square Test 5 as presented by software R.

Test 5	Dataset I	Dataset II	Dataset III	Dataset IV				
	C	T	C	T	C	T	C	T
**Abeli. 1**	n/a	n/a	n/a	n/a	n/a	n/a	n/a	n/a
**Abeli. 2**	n/a	n/a	n/a	n/a	n/a	n/a	n/a	n/a
**χ**^**2**^	0.0746	0.2317	0.0173	0.0461				
**p-value**	1^‡^	1^‡^	1^‡^	1^‡^				
**Fischer’s**	0.71509	0.55326	1.20416	0.73856				
**p-value**	0.8003	0.8611	0.7071	0.8286				

C and T refer to the coastal and terrestrial paleoenvironmental categories, respectively. Numbers after Abeli. (Abelisauridae) represent the taphonomic categories. Positive, negative or lack of any association are signalized by +, -, and n/a, respectively. For the residual analysis values that indicate the type of association see **S1 File**.

^‡^ p-value obtained in Chi-square test using the Monte Carlo analysis.

**Table 7:** Results of Chi-square Test 6 as presented by software R.

Test 6	Dataset I	Dataset II	Dataset III	Dataset IV				
	C	T	C	T	C	T	C	T
**Carch. 1**	n/a	n/a	n/a	n/a	n/a	n/a	n/a	n/a
**Carch. 2**	n/a	n/a	n/a	n/a	n/a	n/a	n/a	n/a
**χ**^**2**^	3.1339	3.0298	2.6228	3.3816				
**p-value**	0.1054^‡^	0.1669^‡^	0.1599^‡^	0.1329^‡^				
**Fischer’s**	0	0	0	0				
**p-value**	0.1033	0.1505	0.1617	0.1282				

C and T refer to the coastal and terrestrial paleoenvironmental categories, respectively. Numbers after Carch. (Carcharodontosauridae) represent the taphonomic categories. Positive, negative or lack of any association are signalized by +, -, and n/a, respectively. For the residual analysis values that indicate the type of association see **S1 File**.

^‡^ p-value obtained in Chi-square test using the Monte Carlo analysis.

**Table 8:** Results of Chi-square Test 7 as presented by software R.

Test 7	Dataset I	Dataset II	Dataset III	Dataset IV				
	C	T	C	T	C	T	C	T
**Spino. 1**	n/a	n/a	n/a	n/a	n/a	n/a	n/a	n/a
**Spino. 2**	n/a	n/a	n/a	n/a	n/a	n/a	n/a	n/a
**χ**^**2**^	3.7607	0.038	3.5979	0.0499				
**p-value**	0.08196^‡^	0.8455^‡^	0.09645^‡^	1^‡^				
**Fischer’s**	3.19102	0.86042	3.8733	1.21006				
**p-value**	0.07777	0.5857	0.1022	0.7505				

C and T refer to the coastal and terrestrial paleoenvironmental categories, respectively. Numbers after Spino. (Spinosauridae) represent the taphonomic categories. Positive, negative or lack of any association are signalized by +, -, and n/a, respectively. For the residual analysis values that indicate the type of association see **S1 File**.

^‡^ p-value obtained in Chi-square test using the Monte Carlo analysis.

**Table 9:** Results of Chi-square Test 8 as presented by software R.

Test 8	Dataset I	Dataset II	Dataset III	Dataset IV				
	C	T	C	T	C	T	C	T
**Early Cretaceous**	n/a	n/a	n/a	n/a	n/a	n/a	n/a	n/a
**Late Cretaceous**	n/a	n/a	n/a	n/a	n/a	n/a	n/a	n/a
**χ**^**2**^	2.2376	5.7445	1.8742	2.056				
**p-value**	0.1347	0.01654*	0.171	0.1516				
**Fischer’s**	1.99268	4.52091	2.35822	2.72479				
**p-value**	0.1739	0.02031*	0.231	0.1955				

C and T refer to the coastal and terrestrial paleoenvironmental categories, respectively. Positive, negative or lack of any association are signalized by +, -, and n/a, respectively. For the residual analysis values that indicate the type of association see **S1 File**.

* Significant p-value.

Each test was performed four times with different datasets regarding the problematic occurrences: 1) “dataset I”, composed by all occurrences; 2) “dataset II”, with possibly paralogous occurrences synonymized; 3) “dataset III”, which lacked the dubious occurrences; 4) “dataset IV”, which excluded the dubious occurrences and contained all possibly paralogous occurrences synonymized (Table 1). These dataset are very different in relation to the number of occurrences. With the exception of the analysis with the Fisher’s exact test, in those cases where the software R presented a warning message for the results, we repeated the Chi-square test choosing the option for the Monte Carlo analysis. The latter is a general term that refers to tests that employ random numbers usually in the form of a computer model (or simulation). The Monte Carlo significance test procedures consist in the comparison between the observed data and random samples generated in accordance with the hypothesis being tested [51]. In other words, we used this method to produce a reference distribution, based on randomly generated samples, which had the same size as the originally tested sample, in order to compute p-values when the Chi-square test requirements were not satisfied. For that, we adopted the R software default parameters [52], which follow Patefield [53]. Finally, the indication of positive or negative associations was obtained by the residual analysis in the Chi-square tests.

As the new approaches applied here represented tests differing in some assumptions, they also tested different hypotheses. So, they are summarized in **S1 Table** according to the hypothesis they tested, the statistics applied (Chi-square or Fisher’s), and the type of table of contingency. For a more practical reference to each test performed with a particular dataset, we will refer to them throughout the text as “test N^F^-X”, where N is the number of the test, the superscript F indicates those analyses with Fisher’s exact test whenever appropriate, and X is the Roman numeral indicative of the used dataset.

## Results

The results are presented below for each test in particular and are summarized in the following tables and **S1 File**.

### Test 1: Taxa and each paleoenvironment separately

Test 1 was performed for each paleoenvironment in particular (Table 2). Regarding the coastal paleoenvironments, only the Chi-square test based on all occurrences, i.e., dataset I (test 1-I), was significant, with a p-value less than 0.05, so rejecting H_0_ and accepting H_1_ –taxa and paleoenvironments are not independent variables. The Chi-square test also showed a positive association between this paleoenvironment and both carcharodontosaurids and spinosaurids, while there was a negative association with abelisaurids. All the other tests, however, obtained p-values greater than 0.05, so they do not reject H_0_ –taxa and paleoenvironments were independent variables and the distribution did not differ significantly from that expected by chance.

On the other hand, all tests for the terrestrial paleoenvironments obtained p-values greater than 0.05, so all of them failed to reject H_0_ –the faunal composition in terrestrial paleoenvironments did not differ significantly from that predicted by simple probability models.

### Test 2: Taxa and both paleoenvironments simultaneously

This test took into account both paleoenvironments simultaneously and only test 2-I obtained a p-value less than 0.05, i.e., H_0_ was rejected (Table 3). In this case the Chi-square test found a positive association between coastal paleoenvironments and both Carcharodontosauridae and Spinosauridae, but a negative one between it and Abelisauridae. However, regarding the terrestrial paleoenvironments, the result was the contrary of the coastal settings: there was a positive association between terrestrial environments and Abelisauridae and a negative one between this environment and both Carcharodontosauridae and Spinosauridae.

### Test 3: Taphonomic categories and each paleoenvironment separately

Here, all tests dealing with coastal occurrences were implemented with Monte Carlo analysis (Table 4). Only tests 3-I and 3-II were significant (p-value less than 0.05), so rejecting H_0_, and they both showed a negative association between this paleoenvironment and taphonomic categories 1 and 2 of Abelisauridae and category 1 of Carcharodontosauridae, whilst it found a positive association with taphonomic category 2 of Carcharodontosauridae and category 2 of Spinosauridae. Regarding category 1 of Spinosauridae, tests obtained different results: 3-I found a positive relationship with coastal paleoenvironments and 3-II, a negative one.

With respect to the terrestrial paleoenvironments, none of the tests needed the Monte Carlo implementation and only tests 3-II and 3-IV obtained p-values greater than 0.05, so failing to reject H_0_. Those significant tests, i.e., rejecting H_0_ and accepting H_1_, obtained different associations between taphonomic categories and terrestrial paleoenvironments. Test 3-I showed a negative association between taphonomic category 1 of all taxa and the paleoenvironment and a positive association for taphonomic category 2 of all taxa. However, with the exclusion solely of dubious occurrences (test 3-III), negative associations were found only between taphonomic category 1 of both Carcharodontosauridae and Spinosauridae and terrestrial paleoenvironments, whereas all other associations were positive.

### Test 4: Taphonomic categories and both paleoenvironments simultaneously

Test 4 was analogous to test 2, but evaluating taphonomic categories (Table 5). Here, only tests 4–1 and 4-III obtained significant results, hence rejecting H_0_ and pointing to a non-random relationship (association) between paleoenvironmental and taphonomic categories. Both significant tests had similar results: coastal paleoenvironments were only positively associated with taphonomic category 2 of Carcharodontosauridae and both categories of Spinosauridae, while the other categories were negatively associated with it; and, terrestrial paleoenvironments were only negatively associated with taphonomic category 2 of Carcharodontosauridae and both categories of Spinosauridae, whilst the other categories were positively associated with it.

### Tests 5 and 5^F^: Abelisaurid taphonomic categories and both paleoenvironments simultaneously

The Chi-square tests implemented with the Monte Carlo analysis found no significant result for all datasets, which did not reject H_0_, i.e., the taphonomic categories of Abelisauridae were randomly distributed within both coastal and terrestrial paleoenvironments (Table 6).

Regarding Fisher’s exact tests (tests 5^F^), all tests obtained p-values greater than 0.05, thus not rejecting H_0_, being similar to the results of test 5.

### Tests 6 and 6^F^: Carcharodontosaurid taphonomic categories and both paleoenvironments simultaneously

Tests 6 for all datasets did not have significant results (Table 7). Thus, H_0_ is still held as valid, which means that taphonomic categories of Carcharodontosauridae and coastal and terrestrial paleoenviroments are independent variables.

With respect to tests 6^F^, all p-values obtained were also greater than 0.05, being not significant and, hence, similar to the results mentioned above.

### Tests 7 and 7^F^: Spinosaurid taphonomic categories and both paleoenvironments simultaneously

Again, all Chi-square tests required the Monte Carlo analysis, but none of them was able to find a significant result, thus not rejecting H_0_ and suggesting a random association between taphonomic categories of Spinosauridae and both paleoenvironments (Table 8). These results were corroborated by the Fisher’s exact tests, which also found only insignificant p-values.

### Tests 8 and 8^F^: Cretaceous epochs and both paleoenvironments simultaneously

Tests 8, in general, obtained p-values greater than 0.05, so not being significant and failing to reject H_0_ (Table 9). This suggests that paleoenvironmental categories are randomly distributed throughout the Cretaceous epochs. Only test 8-II recovered p-values less than 0.05 and showed positive associations between coastal paleoenvironments and the Early Cretaceous epoch and between terrestrial paleoenvironments and the Late Cretaceous epoch. It also found a negative association between coastal paleoenvironments and the Late Cretaceous epoch and between terrestrial paleoenvironments and the Early Cretaceous epoch.

Tests 8^F^ are similar to tests 8 when comparing the results. In general, they also obtained non-significant results, so failing to reject H_0_. Moreover, only test 8^F^-II presented a p-value less than 0.05, and, hence, suggests a non-random relationship between Cretaceous epochs and the compiled number of paleoenvironmental categories.

## Discussion

**Fig 3:**
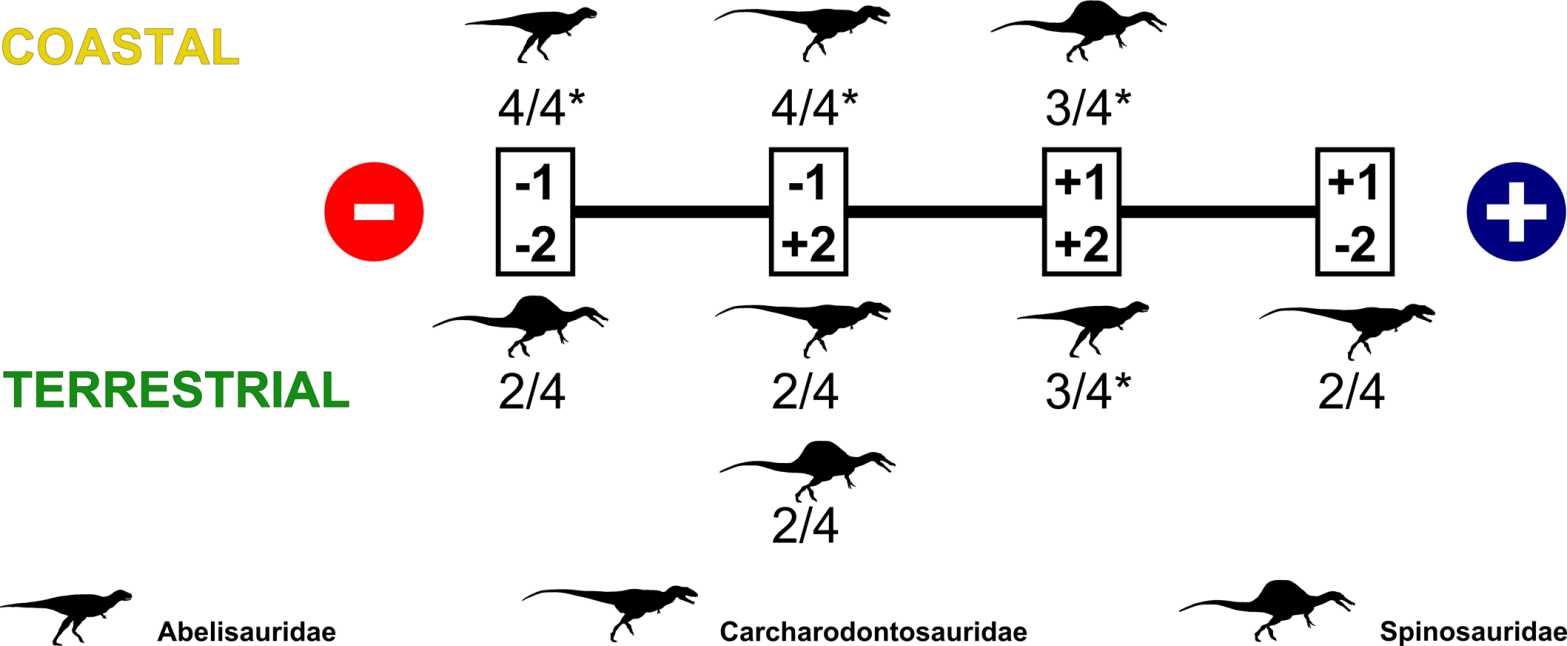
Most frequent associations found between taphonomic categories of each taxon and paleoenvironments in significant tests (3-I, 3-II only for coastal paleoenvironments, 3-III only for terrestrial paleoenvironments, 4-I, and 4-III). The minus and plus signs inside the circles indicate decreasing and increasing trends regarding associations with a particular paleoenvironment, respectively. The rectangles encompass all possible combinations among taphonomic categories and types of association (if negative or positive), which are represented by the numbers and associated signs, respectively. The condition represented by a positively associated category 1 and a negatively associated category 2 (the rightmost rectangle) are closer to an ideal scenario than a real one with respect to the fossil record, as occurrences based on fragmentary records are in general more numerous than those based on more complete specimens. The fractions below the body icons represent the number of times that a given taxon obtained a particular association with a given paleoenvironment (numerator) in relation to the total number of analyses testing this same relationship (denominator). Only associations with a ratio equal or greater than 0.5 are shown, with an asterisk indicating the latter ones.

Keeping in mind the different hypotheses and types of contingency tables, we performed eight different sets of tests, each one possessing its own assumptions (**S1 Table**). So, it is not surprising to find somehow different (and sometimes contrasting) results (Table 10 and Fig 3). Moreover, these tests were supposed to show only if there was or not any non-random relationship between the variables under consideration. In other words, the nature and causes of the presence or absence of non-random relationships are essentially interpretative and require caution to be inferred, especially when the results were divergent. Thus, in order to better compare and discuss our results, this section is divided according to the different aspects we cover both directly and indirectly. When different tests are more similar in their results, we can be more confident about the inferences, while the contrary implies a lower degree of confidence.

**Table 10:** Concordance among tests with significant p-values.

Tests	1-I (C)	2-I	3-I (C)	3-I (T)	3-II (C)	3-III (T)	4-I	4-III
**1-I (C)**	x	3/3	x	x	x	x	x	x
**2-I**	3/3	x	x	x	x	x	x	x
**3-I (C)**	x	x	x	x	5/6	x	6/6	6/6
**3-I (T)**	x	x	x	x	x	5/6	2/6	2/6
**3-II (C)**	x	x	5/6	x	x	x	5/6	5/6
**3-III (T)**	x	x	x	5/6	x	x	3/6	3/6
**4-I**	x	x	6/6	2/6	5/6	3/6	x	12/12
**4-III**	x	x	6/6	2/6	5/6	3/6	12/12	x

C and T refer to paleoenvironments tested alone by tests 1 and 3. The degree of concordance or discordance are indicated by the number of similar associations found by two significant tests in relation to the total number of possible associations tested in common by them. Only tests with similar hypothesis (see **S1 Table**) were compared and when the comparison was not suitable it was indicated by the letter x. Tests with insignificant results were not included due to their promptly recognized agreement among them and disagreement with tests with significant results. As mentioned in the text, all Fisher’s exact tests are also concordant with Chi-square tests for the same datasets, as all of them obtained insignificant p-values, so they were not also included here.

### The implementation of Chi-square tests with the Monte Carlo analysis

We performed Chi-square tests with the Monte Carlo analysis whenever necessary, i.e., in the cases of warning messages given by the software R. To evaluate the reliability of the results obtained with this implementation it is necessary to compare them with the results of other independent approaches, which is the case for tests 5, 5^F^, 6, 6^F^, 7, 7^F^, 8 and 8^F^. In those tests, all contingency tables contained two lines and two columns, and for this type of table Fisher’s exact tests are generally supposed to be the most appropriate statistical analysis [50]. In all of them, Chi-square and Fisher’s approaches obtained similar results for the same datasets regarding their statistical significance (or not) (Tables 6, 7, 8, and 9, and **S1 File**). We consider this as evidence that the Monte Carlo implementation worked well in the sense of not providing unreal significant or non-significant results.

### Effects of problematic occurrences over the different results

Problematic occurrences encompass both possibly paralogous and dubious occurrences. The exclusion of one of these types of occurrences or both was responsible for obtaining different results. This was particularly true for the exclusion of possibly paralogous occurrences (i.e., the synonymization of possibly paralogous occurrences), which was performed in order to diminish possibly untrue overrepresentation of a given taxon in a given environment. Abelisauridae and Spinosauridae were the taxa most affected by this procedure in the sense of obtaining different results when varying the dataset for the tests, the implications of which will be discussed below. The synonymization of possibly paralogous occurrences seemed to affect Abelisauridae especially in tests 3 for terrestrial environments. For instance, we gathered 24 abelisaurid occurrences in Madagascar, but all of them pertain to the Maevarano Formation and are close enough to be considered as only one occurrence after the synonymization procedure (see **S1 Dataset**). On the other hand, the synonymization procedure seemed to affect Spinosauridae especially in tests 3 for coastal settings. Indeed, with respect to paleoenvironments, the coastal ones are those that suffered the greatest relative loss of occurrences by removing the problematic ones and this is clearly exemplified by the results of tests 1, 2, 3, and 4, which in general required the Monte Carlo analysis (Table 2–5 and **S1 File**).

Ideally, the dataset IV was supposed to be the most reliable one for statistical purposes. However, due to the removal of a considerable amount of occurrences, all tests based on it are non-significant and so their results must be seen from a relatively skeptical and comparative point of view instead of considering it alone as providing the most reliable results. We believe that further records along with new findings in the localities listed in this work will reduce the effects of the removal of problematic occurrences over the significance of the results.

### The distribution of paleoenvironments throughout the Cretaceous epochs

Considering the Early and the Late Cretaceous as distinct epochs, we found (except for tests 8-II and 8^F^-II) a random distribution of both coastal and terrestrial paleoenvironments throughout the period. For instance, this implies that the results for Abelisauridae, which had more occurrences in the Late Cretaceous terrestrial paleoenvironments, were more due to the paleoecology of this taxon instead of an uneven distribution of compiled occurrences classified as terrestrial across Cretaceous epochs, which was not the case.

The significant results obtained by tests 8-II and 8^F^-II may be due to the synonymization of paralogous occurrences especially concerning the coastal occurrences. This increased the weight of Early Cretaceous coastal occurrences within the overall period occurrences in relation to the other datasets, which is in accordance with its positive association found by the residual analysis (**S1 File**). Moreover, as none of the Chi-square test 8 required the Monte Carlo analysis, we interpret this as evidence of the reliability of the dataset IV for this test even with a smaller number of occurrences, which, as already mentioned, should be the most reliable dataset. So, tests 8-IV and 8^F^-IV must be the ones that hold the most persuasive results, indicating the lack of association between Cretaceous epochs and paleoenvironmental categories and being contrary to tests 8-II and 8^F^-II.

### Taxa and coastal paleoenvironments

As explained by Butler and Barrett [15], the concept of a taxon being positively and negatively associated with any paleoenvironment is relative to the other taxa sampled. In light of this, tests 1-I and 2-I found a negative association between Abelisauridae and coastal paleoenvironments, but also a positive one between this paleoenvironment and both Carcharodontosauridae and Spinosauridae. Tests with other datasets did not obtain significant results, probably due to the reduced number of occurrences in relation to dataset I. The significant result mentioned above could suggest that both carcharodontosaurids and spinosaurids were more distributed in coastal paleoenvironments than abelisaurids in a way that differed significantly from that predicted by simple probability models.

**Fig 4:**
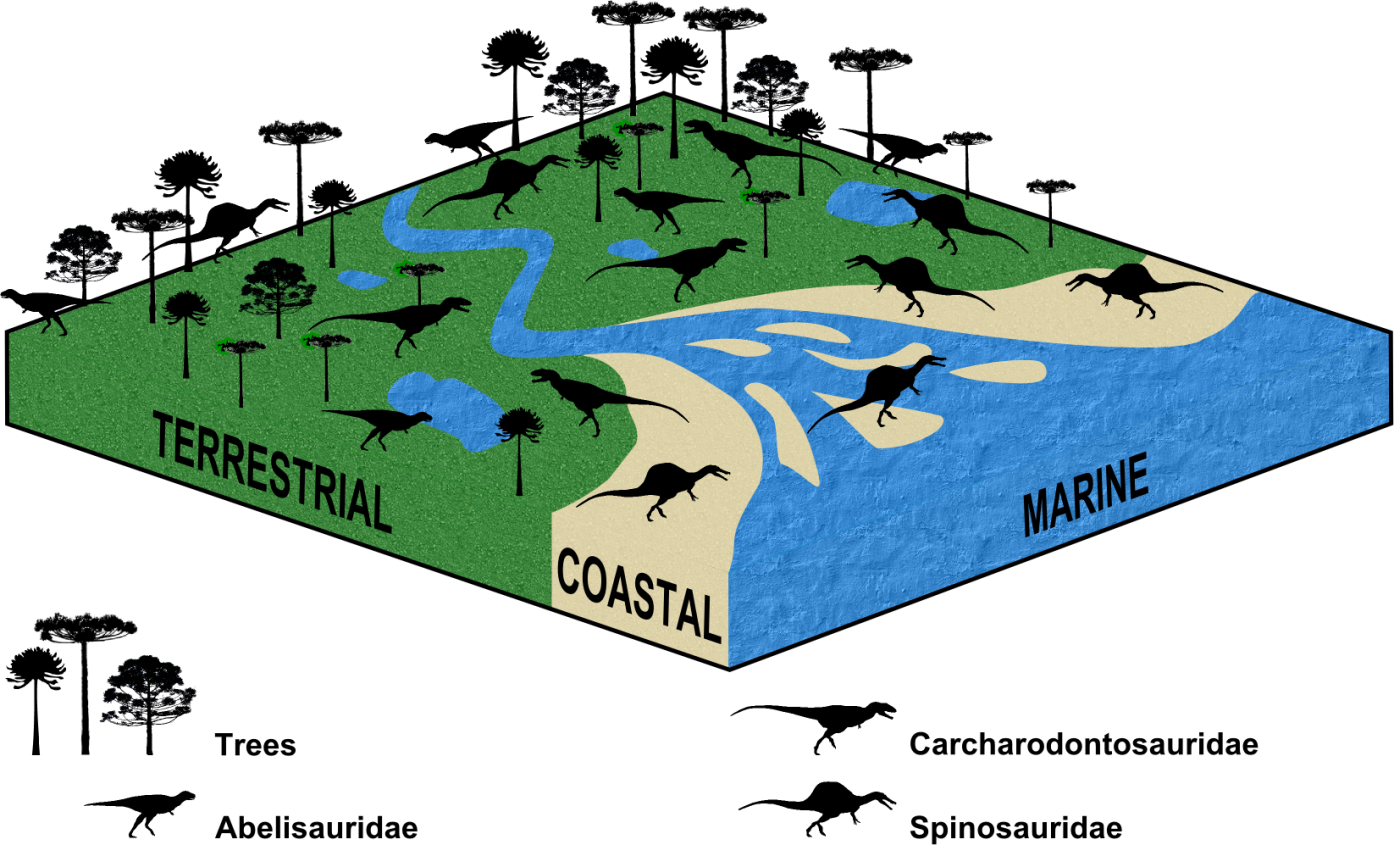
Schematic illustration of the spatial distribution of Abelisauridae, Carcharodontosauridae, and Spinosauridae throughout coastal and terrestrial paleoenvironments. Spinosaurids seem to have been natural inhabitants of coastal settings, while terrestrial and more inland habitats were shared by them and both abelisaurids and carcharodontosaurids. Note that the number of body icons (not to scale) does not reflect perfectly the relative abundance of these taxa within each paleoenvironment.

Nevertheless, test 3-I and 3-II showed that all taphonomic categories of Abelisauridae and category I of Carcharodontosauridae were negatively associated with coastal paleoenvironments, while category 2 of Carcharodontosauridae and Spinosauridae were positively associated. In addition, Test 3-I found a positive association also between category 1 of Spinosauridae and coastal areas. Tests 4-I and 4-III also obtained significant results, being similar to those of test 3-I (Table 10). These results suggest that, despite the number of occurrences of Carcharodontosauridae in coastal sediments being statistically more comparable to that of Spinosauridae than to Abelisauridae, the fossil record of carcharodontosaurids in this paleoenvironment is basically composed by fragmentary and isolated specimens, which points to a higher degree of transportation, whereas spinosaurids in coastal sediments are represented by more complete specimens, implying that they suffered significantly lower degrees of transportation (Fig 3). As the presence of fragmented and disarticulated fossil remains is more expected for distal sedimentary systems like deltas and coastal settings, which was the case for carcharodontosaurids and abelisaurids, the presence of articulated remains in these settings implies that spinosaurids were “truly” occupying coastal environments or at least habitats close by, while the other theropods in general had more inland habitats (Fig 4). It is likely that there were also some differences between abelisaurids and carcharodontosaurids, with the former inhabiting (or being more common in) even more inland areas than the latter, which could explain the negative association of category 2 of the abelisaurids and the positive one of category 2 of carcharodontosaurids with coastal paleoenvironments. If it was the case for species with overlapping geographic ranges, so abelisaurids and carcharodontosaurids might have been sympatric, but not exactly syntopic [11].

### Taxa and terrestrial paleoenvironments

Discussing the type of association between those taxa and terrestrial paleoenvironments is a more complicated issue given the different results for some of them. Test 1 obtained no significant result for all datasets, suggesting that no taxon was significantly more distributed in inland areas than the others. Here may be one of the few cases where results based on datasets II, III, and IV are more reliable, because the number of occurrences in this paleoenvironment were still high despite the exclusion of paralogous and dubious occurrences. Actually, considering the large number of possibly paralogous occurrences of Malagasy abelisaurids, it would be more cautious to base inferences on these datasets instead of only on dataset I as in the other tests mentioned above.

However, test 2 differed somehow from test 1. Test 2-I found a positive association between Abelisauridae and terrestrial paleoenvironments, whilst both Carcharodontosauridae and Spinosauridae were negatively associated with them. Test 2 with other datasets found no significant result. As test 2 took into account both coastal and terrestrial paleoenvironments, the non-significance of some results may be due to two different factors. The first one is the removal of problematic coastal occurrences, which may be responsible for not finding any positive or negative relationship of any taxa with coastal environments, as explained before. The second factor is that the synonymization of possibly paralogous occurrences made the number of occurrences in terrestrial environments of each taxon more similar in relation to the others, and, if this was the case, so observations made above for test 1 might be also valid for test 2. On the other hand, the significance of test 2-I may be more due to the coastal occurrences than exactly the number of terrestrial occurrences attributable for each clade.

Given the statements above, the comparisons between the results of tests 1 and 2 seem to point in general for the absence of any taxon significantly associated with terrestrial paleoenvironments, be it a positive or negative association. This makes sense when considering that abelisaurids and carcharodontosaurids were in general considered essentially terrestrial, and the positive association of the first and the negative association of the latter were possibly the result of the inflated number of Malagasy abelisaurid possibly paralogous occurrences in terrestrial sediments and the higher number of carcharodontosaurid occurrences in coastal areas, instead of a more widespread distribution of abelisaurids in inland areas. An alternative is that abelisaurids, independently of being or not more widespread, could have been more numerous in inland habitats than carcharodontosaurids and this could have enhanced the potential for preserving more remains of the former in relation to the latter (and also spinosaurids; see below).

However, the fact that the occurrences of Spinosauridae in terrestrial paleoenvironments are not significantly different from those of Abelisauridae and Carcharodontosauridae may be somehow surprising, and understanding the real meaning of this result in particular requires a taphonomic approach. Test 3 found three different results. As already mentioned, the type of association between taphonomic categories of Abelisauridae and terrestrial paleoenvironments varies according to the dataset used (see the residual analysis in **S1 File**). On the other hand, where the results were significant (using datasets I and III), the association between taphonomic categories and this paleoenvironment were similar for both Carcharodontosauridae and Spinosauridae: category 1 being negatively associated and category 2 positively associated (Table 4 and Fig 3). As these taphonomic categories are supposed to be related to transportation, these similarities between carcharodontosaurids and spinosaurids might suggest they occupied spatially inland habitats in a way more similar than usually depicted. This is also supported by tests 3-II and 3-IV, which found no significant association between terrestrial paleoenvironments and the taphonomic categories of any clade.

On the other hand, test 4 found positive associations between all categories of Abelisauridae and terrestrial sediments when the p-values were significant (using datasets I and III). It also obtained positive associations between the latter and category 1 of Carcharodontosauridae, while categories 2 of carcharodontosaurid and both taphonomic categories of Spinosauridae were negatively associated. These results contrast with those of test 3, suggesting distinct ways of occupation of inland habitats for carcharodontosaurids and spinosaurids (Fig 3). However, the observations made for test 2 also apply to test 4 with respect to the effects of few occurrences in coastal sediments especially for Abelisauridae and Carcharodontosauridae, as test 4 also analyzed both paleoenvironments simultaneously. For instance, all tests that included coastal occurrences of dataset I and analyzed all taxa simultaneously obtained significant p-values.

It may be now appropriate to take into consideration the results of tests 5, 5^F^, 6, 6^F^, 7, and 7^F^. As they analyzed each taxon in particular, they might reflect the statistical behavior of the taphonomic categories in relation to each paleoenvironment without the interference of the number of occurrences of other taxa. All tests had non-significant results. This could be a consequence of the low number of occurrences in coastal environments along with the removal of problematic occurrences in the cases of datasets other than dataset I, but these results may be somehow logical. For instance, spinosaurids are supposed to have had semi-aquatic lifestyles and a probable preference for marginal habitats [7, 16, 19, 20], which were not restricted to coastal areas: they may also have inhabited river and lake margins located more inland. If this was the case, it could be speculated that the taphonomic categories should behave statistically in a similar way for both coastal and terrestrial paleoenvironments, instead of the results shown by tests 4-I and 4-III, which took into account the relative amount of occurrences of the other taxa. It could be also a consequence of the way we defined the taphonomic categories and the types of problematic occurrences, which were a direct consequence of the amount of available data, being, in turn, related to collection efforts. New findings may increase the number of occurrences for each category, reduce the number of problematic ones (especially those dubious), and even enable a more refined definition of the taphonomic and paleoenvironmental categories. This reasoning is applicable to all taxa.

At this time, considering all the available information, it is not possible to rule out the hypothesis of spinosaurids occupying spatially inland habitats in a way somehow similar to that of other terrestrial taxa, like carcharodontosaurids (Figs 3 and 4). This implies that spinosaurids could have been more generalist or at least less specialized than usually suggested regarding types of habitats, being well represented in from coastal to inland areas ([7] contra [21]). Also, the differences observed between the taphonomic categories of Abelisauridae and Carcharodontosauridae may be more due to the greater number of problematic (especially possibly paralogous) occurrences of Abelisauridae than to real and disparate differences regarding the spatial niche of these two taxa. However, the possibility of abelisaurids having been more numerous within continental settings than carcharodontosaurids may also have played a role in this regard, which could explain the association found for the category 1 of each taxa (the most abundant taxon would have more potential to have a fossil record composed by more complete specimens).

### Other paleoecological implications

Differences regarding the number of occurrences among the three taxa could be first seen by some as an indirect measurement of their relative abundance. In fact, Hone et al. [48] suggested that spinosaurids could have been rare animals when compared to other theropod taxa with larger fossil record, like tyrannosaurids and allosaurids, a view somehow opposed by that of Benyoucef et al. [21]. However, as our work dealt primarily with locations and as each location may be the source of more than one fossil specimen, our compilation of occurrences had more to do with large-scale geographic distribution than with relative abundance. However, as taphonomic category 1 is based on more complete and better preserved specimens and some localities yielded only one specimen, it can be used as indirect information on the minimum number of at least this type of record for each clade. Category 2, on the other hand, may be less informative because it is based on isolated and fragmentary remains, encompassing mainly isolated teeth. The number of occurrences classified into category 1 is 27, 15, and 18 for Abelisauridae, Carcharodontosauridae, and Spinosauridae, respectively. Despite the number of other skeletal materials eventually classified into category 2 and one special case of category 1 of Thai spinosaurids (see below), our dataset shows that the number of spinosaurid skeletal materials is not considerably fewer than that of the other taxa–actually, category 1 of spinosaurids is more numerous than category 1 of carcharodontosaurids.

Given the number of skeletal materials for tyrannosaurids and allosaurids mentioned by Hone et al. [48], if one assumes spinosaurids as rare faunal components, the same inference must be applied to carcharodontosaurids and possibly to abelisaurids, depending on the adopted concept of rareness. However, it is unlikely that all these taxa were rare components in comparison to other medium to large-bodied theropod clades, although they would be minor components of local paleofaunas in comparison to sympatric small-bodied theropods, as proposed for tyrannosaurids [54, 55]. Indeed, as suggested above, some differences may have existed between the abundance of abelisaurids and carcharodontosaurids, with the former being more numerous, as expected due to their difference in body size [11]–carcharodontosaurids were bigger, although some taxa overlap in size with abelisaurids–and roughly indicated by the number of category 1 occurrences of each taxa and their behavior in tests 3-I, 3-III, 4–1, and 4-III regarding terrestrial paleoenvironments.

Most occurrences of Abelisauridae, Carcharodontosauridae, and Spinosauridae are Gondwanan, and collection efforts in Africa and South America in general are considerably smaller than in Asia and North America, from which came most of the fossil record of tyrannosaurids and allosaurids. Moreover, many specimens of tyrannosaurids and allosaurids came from bonebeds, some of them being monodominant assemblages in some outcrops, which may be related to social behavior and mass death events [11, 54, 56]. Bonebeds containing Jurassic and Cretaceous large theropods are quite rare in Gondwana. Actually, Laje do Coringa in the Northeastern Brazil is one of such sites, but its fossils are very fragmentary as they seem to have been reworked. Not surprisingly, the most common theropod remains there are teeth, and the only theropod cranial material from this site reported so far are the holotypic premaxillae (and a partial maxilla) of the spinosaurine spinosaurid *Oxalaia quilombensis*. As the Laje do Coringa bonebed is supposed to have been formed within a coastal setting, it is more probable that *O*. *quilombensis* was a species that “truly” inhabited this paleoenvironment, while the abundant teeth of carcharodontosaurids might have come from further inland areas [25, 57, 58].

Hone et al. [48] also commented that a taphonomic bias in favor of spinosaurid remains in the fossil record in comparison to other theropod taxa should be expected, as the former might have inhabited preferentially aquatic habitats. However, the rareness of spinosaurid specimens would suggest the contrary, and this could correlate with the rareness of these theropods in the fossil record, although the most abundant dinosaur remains in the Romualdo Formation are those identified as spinosaurids [59–64]. Besides, they also suggest that spinosaurids should have been present also in North America (see also [65]) and their absence in the North American fossil record should be considered as evidence further corroborating the hypothesis of their scarcity in paleoecosystems. Actually, the evidence for the presence of spinosaurids in North America during the Late Jurassic is dubious (**S1 Dataset**) and the hypothesis of rareness requires much more evidence. Interestingly, some of our results suggest that the fossil records in terrestrial sediments of spinosaurids and carcharodontosaurids are equivalent, which points to some similarity regarding the spatial distribution across inland habitats, as mentioned earlier.

**Fig 5:**
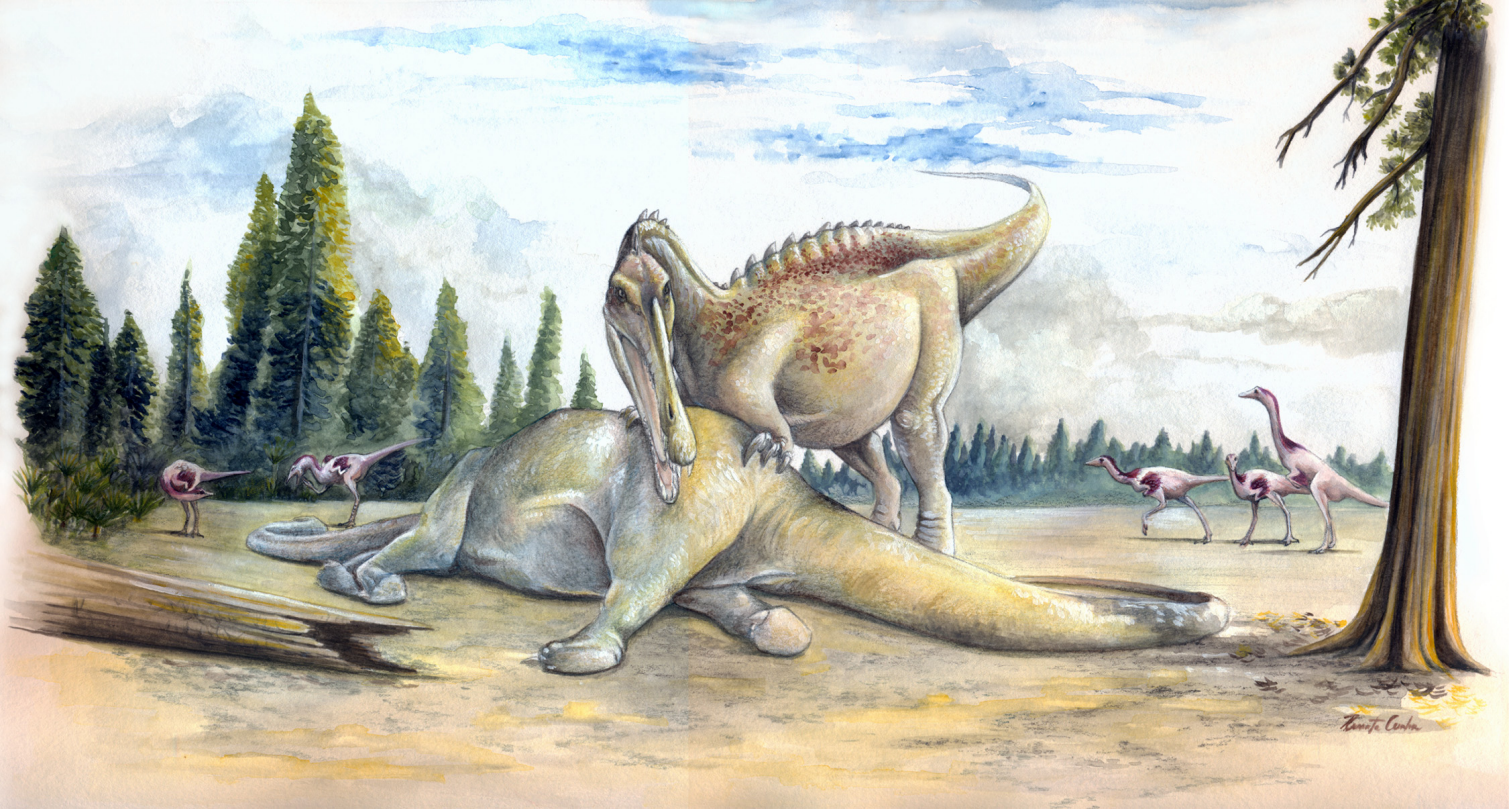
Reconstruction of the terrestrial paleoenvironmental setting of the Sao Khua Formation. In the center, a generalized spinosaurid feeds on a sauropod. This trophic relationship is hypothesized based on isolated tooth crowns found in association with a sauropod skeleton [67]. In the background, a small pack of the ornithomimosaur theropod *Kinnareemimus*. Both sauropods and ornithomimosaurs (as part of the “herbivorous” theropods) were found to be positively associated with terrestrial paleoenvironments by Butler and Barrett [15].

Some might consider this hypothesis as unlikely, especially after the work of Ibrahim et al. [20] on new materials attributed to the spinosaurine spinosaurid *Spinosaurus aegyptiacus* from the Kem Kem beds of Morocco. Benyoucef et al. [21] also discussed the vertebrate fossil record of the Mid-Cretaceous Saharan deposits, pointing the overabundance of spinosaurids in some Algerian localities and suggesting that this taxon preferentially inhabited environments with few plant-eating dinosaurs and close to the seashore. However, an association with a particular habitat may have more to do with avoidance of other competitors or predators than with a real preference for it, and this is especially difficult to be tested regarding the fossil record (see examples in Farlow and Pianka [11]). Furthermore, our test 4 could not rule out the inference of spinosaurids being able to inhabit also inland or non-marginal paleoenvironments. Additionally, our dataset also comprises species other than *S*. *aegyptiacus*, which clearly had body plans different from the one proposed by Ibrahim et al. [20]. In fact, despite of all spinosaurid taxa possessing anatomical features indicative of semi-aquatic lifestyles or a mostly piscivorous diet, there is also evidence suggesting that these theropods could have behaved more plastically than usually inferred. The fossil record of predation by spinosaurids indicates that they also included other animals in their diet. Spinosaurids seem to have fed also on iguanodontids, pterosaurs, and sauropods [16, 62, 66, 67], and the latter were found to be positively associated with terrestrial paleoenvironments (and negatively associated with the coastal ones) by Butler and Barrett [15]. In fact, the Thai record of predation of sauropods by spinosaurids comes from a paleoecological setting interpreted as terrestrial (Fig 5; **S1 Dataset**) [66, 67] and this could also explain the rareness of herbivorous dinosaurs in coastal localities with spinosaur remains, as the most common herbivores of the Mid-Crateceous of Gondwana were sauropods [27, 28]. Moreover, Therrien et al. [68] found a mandibular force profile for the baryonychine spinosaurid *Suchomimus tenerensis* that would have made it able to feed on small to medium-size terrestrial prey, whereas the robust forelimbs could have been used for hunting large ones [69]. Also, the same study that found isotopic values indicative of semi-aquatic lifestyles for spinosaurids also reported some values comparable to those of terrestrial taxa, including specimens from Morocco [7]. So, in short, the taphonomic evidence showed that spinosaurids also inhabited distal sedimentary environments, although clearly not exclusively or even mostly.

Occupying more inland areas may have compensated for the supposed bias favoring the preservation of spinosaurid remains (where it is not observed) and this hypothesis has also other paleoecological implications. Although the proposition of extinction by the destruction of coastal habitats due to the increase of sea levels is specific for *Spinosaurus* and *Carcharodontosaurus* [22], one might extend this for their respective families as a whole. However, as shown above, carcharodontosaurids were more associated with terrestrial habitats, whilst spinosaurids were also present in more inland areas. So, the rise of sea level may not explain the extinction of these taxa during the early Late Cretaceous in Gondwana. Besides, the rise of sea level affects more strongly the inland fauna, as coastal environments migrate backwards into the continent, diminishing the habitable areas. Actually, the possible occurrences of these taxa in more young deposits located both inside and outside Gondwana (**S1 Dataset**) (e.g., [38, 48, 70]) further complicate this scenario, and different causes acting in different settings and moments may have been responsible for the extinction of these clades in each region in particular [29]. Once extinct, carcharodontosaurids and, possibly, spinosaurids were replaced by abelisaurids–whose fossil record is trackable until the Late Jurassic [71]–as the top predators of inland areas especially in Gondwana [28, 29]. Although megaraptoran and unenlagiid theropods are also thought to have played an important role as medium to large-size predators, we were not able to include them in our analysis due to the small number of occurrences. However, we hope this will change in the near future due to the new findings that have been reported continuously [29, 72].

## Final Remarks

Our study aimed to evaluate statistically the relationship between Abelisauridae, Carcharodontosauridae, and Spinosauridae and coastal and terrestrial paleoenvironments. In short, our results are: 1) spinosaurids were the taxon with the largest amount of statistical evidence showing it positively associated with coastal paleoenvironments; 2) abelisaurids and carcharodontosaurids were more associated with terrestrial paleoenvironments; 3) some of our results support the idea of spinosaurids also inhabiting inland areas, being comparable at least to carcharodontosaurids regarding the spatial distribution throughout this paleoenvironment; 4) abelisaurids could have been more numerous than carcharodontosaurids and, possibly, spinosaurids in inland habitats.

They also point to some practical details pervading this sort of analysis. Some fossil sites may represent the same paleoenvironment, and, in this case, they play a significant role in the statistical significance of some results. So, it is useful to address this sort of bias in order to have a more complete appreciation of the robustness of the positive or negative (if any) associations recovered. Chi-square tests implemented with the Monte Carlo analysis also seemed to have worked well, due to the coherence between the results found using it and those of Fisher’s exact test, so they can be used in further analysis. Also, classifying occurrence in taphonomic categories enabled further refinements of the nature of the associations eventually found by our tests. Although these new approaches make some of our tests not exactly similar to the ones performed by Butler and Barrett [15], we consider our methodology as complementary to the latter.

We are aware that our results are valid within the assumptions stated here–from our conceptual framework to our geological and taxonomic assignments–and until new occurrences are included in our datasets, and, so, all inferences made by us can be seen as hypothesis for further tests. This will be necessary when new records change the absolute number of occurrences of the taphonomic categories or each paleoenvironment and we hope this happens as science is replicable in essence. Actually, this is already the case, as our deadline for gathering published references was December 31, 2014 and new discoveries have been reported since then (e.g., [21, 73, 74]). However, it is not one, two, or three new findings that will change the validity of our results, especially when they come from localities already recorded in our datasets for each clade and/or do not change the taphonomic categories in which a given occurrence was classified. Furthermore, our results can be reappraised also by changing some of our concepts, like those regarding problematic occurrences or taphonomic categories. One can also change the classification of the occurrences into the broad paleoenvironmental categories in case of disagreement with our propositions. In fact, new discoveries may be also responsible for this re-evaluation. Finally, a hypothesis is more robust when different approaches support it, thus the propositions here presented may be strengthened by new analyses with other methodological bases and data.

## Supporting Information

S1 Appendix Data collection through Fossilworks webpage. (RTF)

S1 Dataset List of occurrences. (XLSX)

S1 File Results of Chi-square and Fisher’s exact tests, including residual analysis. The residual analysis is presented for each taxon and paleoenvironment as the numbers within the cells.
(XLSX)

S1 Letter Terms of image use. (PDF)

S1 Table Summarization of the main features of each statistical test performed. (RTF)
