# Correction: The "*χ*" of the Matter: Testing the Relationship between Paleoenvironments and Three Theropod Clades

**DOI:** 10.1371/journal.pone.0152634

**Published:** 2016-03-28

**Authors:** The PLOS ONE Staff

Due to a typesetting error, the headings in Tables 1–9 appear incorrectly. The publisher apologizes for the errors. Please see the corrected tables here.

**Table 1:** Different datasets of occurrences.

Taxon or Period	Taphonomic Category or Epoch	Dataset I		Dataset II		Dataset III		Dataset IV	
		C	T	C	T	C	T	C	T
**Abeli. (72)**	**1**	1	26	1	12	1	24	1	11
	**2**	2	37	2	13	1	29	1	8
	**Not specified**	0	0	0	0	0	0	0	0
**Carch. (66)**	**1**	0	15	0	11	0	15	0	10
	**2**	9	41	6	20	5	27	5	13
	**Not specified**	1	0	1	0	0	0	0	0
**Spino. (82)**	**1**	6	12	3	10	5	10	3	8
	**2**	8	52	7	20	4	32	4	13
	**Not specified**	2	1	1	1	0	0	0	0
**Cretaceous (194)**	**Early**	14	77	9	34	10	54	8	27
	**Late**	8	88	4	36	3	74	3	28

C and T refer to the costal and terrestrial paleoenvironmental categories, respectively, while taxa are indicated by Abeli. (Abelisauridae), Carch. (Carcharodontosauridae), and Spino. (Spinosauridae). On the other hand, taphonomic categories are indicated by their respective numbers except for those occurrences lacking data about the nature of their fossil record, whose taphonomic categories were considered as not specified. Within brackets is the total number of occurrences of each taxon or period.

**Table 2:** Results of Chi-square Test 1 as presented by software R.

Test 1	Dataset I		Dataset II		Dataset III		Dataset IV	
	C	T	C	T	C	T	C	T
**Abeli.**	-	n/a	n/a	n/a	n/a	n/a	n/a	n/a
**Carch.**	+	n/a	n/a	n/a	n/a	n/a	n/a	n/a
**Spino.**	+	n/a	n/a	n/a	n/a	n/a	n/a	n/a
**χ^2^**	8.7586	0.7283	4.5714	0.8276	4.625	1.7664	2.7143	0.381
**p-value**	0.01253*	0.6948	0.1017	0.6611	0.09901	0.4135	0.2894^‡^	0.8266

C and T refer to the coastal and terrestrial paleoenvironmental categories, respectively, while taxa are indicated by Abeli. (Abelisauridae), Carch. (Carcharodontosauridae), and Spino. (Spinosauridae). Positive, negative or lack of any association are signalized by +, -, and n/a, respectively. For the residual analysis values that indicate the type of association see S1 File.

**Table 3:** Results of Chi-square Test 2 as presented by software R.

Test 2	Dataset I		Dataset II		Dataset III		Dataset IV	
	C	T	C	T	C	T	C	T
**Abeli.**	-	+	n/a	n/a	n/a	n/a	n/a	n/a
**Carch.**	+	-	n/a	n/a	n/a	n/a	n/a	n/a
**Spino.**	+	-	n/a	n/a	n/a	n/a	n/a	n/a
**χ**^**2**^	7.3431		2.6081		5.5498		1.9352	
**p-value**	0.02544*		0.2714		0.07246^‡^		0.4308^‡^	

C and T refer to the coastal and terrestrial paleoenvironmental categories, respectively, while taxa are indicated by Abeli. (Abelisauridae), Carch. (Carcharodontosauridae), and Spino. (Spinosauridae). Positive, negative or lack of any association are signalized by +, -, and n/a, respectively. For the residual analysis values that indicate the type of association see **S1 File**.

* Significant p-value.

^‡^ p-value obtained in Chi-square test using the Monte Carlo analysis.

**Table 4:** Results of Chi-square Test 3 as presented by software R.

Test 3	Dataset I		Dataset II		Dataset III		Dataset IV	
	C	T	C	T	C	T	C	T
**Abeli. 1**	-	-	-	n/a	n/a	+	n/a	n/a
**Abeli. 2**	-	+	-	n/a	n/a	+	n/a	n/a
**Carch. 1**	-	-	-	n/a	n/a	-	n/a	n/a
**Carch. 2**	+	+	+	n/a	n/a	+	n/a	n/a
**Spino. 1**	+	-	-	n/a	n/a	-	n/a	n/a
**Spino. 2**	+	+	+	n/a	n/a	+	n/a	n/a
**χ^2^**	16.9231	39.918	12.2632	7.0698	9.5	16.0657	8.2857	2.4286
**p-value**	0.0065*^‡^	1.55E-07*	0.03698*^‡^	0.2155	0.09845^‡^	0.006659*	0.1554^‡^	0.7872

C and T refer to the coastal and terrestrial paleoenvironmental categories, respectively, while taxa are indicated by Abeli. (Abelisauridae), Carch. (Carcharodontosauridae), and Spino. (Spinosauridae). Numbers after the taxa represent the taphonomic categories. Positive, negative or lack of any association are signalized by +, -, and n/a, respectively. For the residual analysis values that indicate the type of association see **S1 File**.

* Significant p-value.

^‡^ p-value obtained in Chi-square test using the Monte Carlo analysis.

**Table 5:** Results of Chi-square Test 4 as presented by software R.

Test 4	Dataset I		Dataset II		Dataset III		Dataset IV	
	C	T	C	T	C	T	C	T
**Abeli. 1**	-	+	n/a	n/a	-	+	n/a	n/a
**Abeli. 2**	-	+	n/a	n/a	-	+	n/a	n/a
**Carch. 1**	-	+	n/a	n/a	-	+	n/a	n/a
**Carch. 2**	+	-	n/a	n/a	+	-	n/a	n/a
**Spino. 1**	+	-	n/a	n/a	+	-	n/a	n/a
**Spino. 2**	+	-	n/a	n/a	+	-	n/a	n/a
**χ**^**2**^	14.6137		5.3791		13.8029		5.3592	
**p-value**	0.01249*^‡^		0.3923^‡^		0.01549*^‡^		0.3758^‡^	

C and T refer to the coastal and terrestrial paleoenvironmental categories, respectively, while taxa are indicated by Abeli. (Abelisauridae), Carch. (Carcharodontosauridae), and Spino. (Spinosauridae). Numbers after the taxa represent the taphonomic categories. Positive, negative or lack of any association are signalized by +, -, and n/a, respectively. For the residual analysis values that indicate the type of association see **S1 File**.

* Significant p-value.

^‡^ p-value obtained in Chi-square test using the Monte Carlo analysis.

**Table 6:** Results of Chi-square Test 5 as presented by software R.

Test 5	Dataset I		Dataset II		Dataset III		Dataset IV	
	C	T	C	T	C	T	C	T
**Abeli. 1**	n/a	n/a	n/a	n/a	n/a	n/a	n/a	n/a
**Abeli. 2**	n/a	n/a	n/a	n/a	n/a	n/a	n/a	n/a
**χ^2^**	0.0746		0.2317		0.0173		0.0461	
**p-value**	1^‡^		1^‡^		1^‡^		1^‡^	
**Fischer’s**	0.71509		0.55326		1.20416		0.73856	
**p-value**	0.8003		0.8611		0.7071		0.8286	

C and T refer to the coastal and terrestrial paleoenvironmental categories, respectively. Numbers after Abeli. (Abelisauridae) represent the taphonomic categories. Positive, negative or lack of any association are signalized by +, -, and n/a, respectively. For the residual analysis values that indicate the type of association see **S1 File**.

^‡^ p-value obtained in Chi-square test using the Monte Carlo analysis.

**Table 7:** Results of Chi-square Test 6 as presented by software R.

Test 6	Dataset I		Dataset II		Dataset III		Dataset IV	
	C	T	C	T	C	T	C	T
**Carch. 1**	n/a	n/a	n/a	n/a	n/a	n/a	n/a	n/a
**Carch. 2**	n/a	n/a	n/a	n/a	n/a	n/a	n/a	n/a
**χ^2^**	3.1339		3.0298		2.6228		3.3816	
**p-value**	0.1054^‡^		0.1669^‡^		0.1599^‡^		0.1329^‡^	
**Fischer’s**	0		0		0		0	
**p-value**	0.1033		0.1505		0.1617		0.1282	

C and T refer to the coastal and terrestrial paleoenvironmental categories, respectively. Numbers after Carch. (Carcharodontosauridae) represent the taphonomic categories. Positive, negative or lack of any association are signalized by +, -, and n/a, respectively. For the residual analysis values that indicate the type of association see **S1 File**.

^‡^ p-value obtained in Chi-square test using the Monte Carlo analysis.

**Table 8:** Results of Chi-square Test 7 as presented by software R.

Test 7	Dataset I		Dataset II		Dataset III		Dataset IV	
	C	T	C	T	C	T	C	T
**Spino. 1**	n/a	n/a	n/a	n/a	n/a	n/a	n/a	n/a
**Spino. 2**	n/a	n/a	n/a	n/a	n/a	n/a	n/a	n/a
**χ^2^**	3.7607		0.038		3.5979		0.0499	
**p-value**	0.08196^‡^		0.8455^‡^		0.09645^‡^		1^‡^	
**Fischer’s**	3.19102		0.86042		3.8733		1.21006	
**p-value**	0.07777		0.5857		0.1022		0.7505	

C and T refer to the coastal and terrestrial paleoenvironmental categories, respectively. Numbers after Spino. (Spinosauridae) represent the taphonomic categories. Positive, negative or lack of any association are signalized by +, -, and n/a, respectively. For the residual analysis values that indicate the type of association see **S1 File**.

^‡^ p-value obtained in Chi-square test using the Monte Carlo analysis.

**Table 9:** Results of Chi-square Test 8 as presented by software R.

Test 8	Dataset I		Dataset II		Dataset III		Dataset IV	
	C	T	C	T	C	T	C	T
**Early Cretaceous**	n/a	n/a	n/a	n/a	n/a	n/a	n/a	n/a
**Late Cretaceous**	n/a	n/a	n/a	n/a	n/a	n/a	n/a	n/a
**χ^2^**	2.2376		5.7445		1.8742		2.056	
**p-value**	0.1347		0.01654*		0.171		0.1516	
**Fischer’s**	1.99268		4.52091		2.35822		2.72479	
**p-value**	0.1739		0.02031*		0.231		0.1955	

C and T refer to the coastal and terrestrial paleoenvironmental categories, respectively. Positive, negative or lack of any association are signalized by +, -, and n/a, respectively. For the residual analysis values that indicate the type of association see **S1 File**.

* Significant p-value.
