# Supplementary material for: The “χ” of the Matter: Testing the Relationship between Paleoenvironments and Three Theropod Clades
Source: PLoS One. 2016 Feb 1;11(2):e0147031. doi: 10.1371/journal.pone.0147031 (PMC4734717; doi:10.1371/journal.pone.0147031)
Supplement: S1 Appendix — (RTF) [file pone.0147031.s001.rtf]

Data collection through Fossilworks webpage
Fossilworks [1] is an access link to the Paleobiology Database (PaleoDB), a vast compilation of data on fossils, like geographic and stratigraphic provenance, references, and classification opinions. Thus it provides a valuable tool for gathering data for large-scale studies, especially those focused on macroevolutionary patterns, like that of Butler and Barrett [2].
This webpage offers many tools for accessing PaleoDB. For downloading occurrences, it is necessary to fill an online form defining the type of data required, like the taxonomic level and time intervals. However, in this case, there are only two options of taxonomic level – species and genus. Early attempts of compiling occurrences of Abelisauridae, Carcharodontosauridae, and Spinosauridae by following that procedure revealed to be biased by the exclusion of those occurrences based on specimens attributed to suprageneric taxonomic levels [3-5].
In order to optimize data collection we chose not to follow the standard procedure highlighted above. We employed the search tool called “Fossil collection records” for gathering occurrence data. We did so because the other available search tool for our purposes – “Fossil organisms” – seemed to be less efficient in compiling all occurrences attributed to a particular taxon, i.e., it eventually indicated fewer occurrences than “Fossil collection records”. However, it is important to mention that even this method was unable to present all occurrences recorded in PaleoDB, which was realized when consulting published references later. 
We performed searches for each taxon at a time. The occurrences and their associated data were then manually recorded in Excel (S2 Dataset). The deadline for gathering data through both PaleoDB and published references not included in PaleoDB was December 31, 2014. References after this date, like Gianechini et al. [6] and Benyoucef et al. [7], were not included, although the first one was took into account for discussing the statistical results.
The collected data was then compared to other references [e.g., 8-12] for checking their validity and evaluating the taxonomic and paleoenvironmental assignments. Thus, our list of occurrences for each taxon differs from that of PaleoDB. Also, our paleoenvironmental interpretations differ somehow from those of both PaleoDB and Butler and Barrett [2] (S2 Dataset).

References
1. Fossilworks. 2015. Available: http://fossilworks.org/.
2. Butler RJ, Barrett PM. Palaeoenvironmental controls on the distribution of Cretaceous herbivorous dinosaurs. Naturwissenschaften. 2008; 95: 1027-1032. doi: 10.1007/s00114-008-0417-5
3. Carrano MT, Butler RJ, Mannion PD. Taxonomic occurrences of Abelisauridae recorded in the Paleobiology Database. Fossilworks. 2014. Available: http://fossilworks.org.
4. Carrano MT, Alroy J, Benson RBJ, Mannion, PD. Taxonomic occurrences of Carcharodontosauridae recorded in the Paleobiology Database. Fossilworks. 2014. Available: http://fossilworks.org.
5. Carrano MT, Alroy J, Benson RBJ, Lloyd GT, Mannion PD. Taxonomic occurrences of Spinosauridae recorded in the Paleobiology Database. Fossilworks. 2014. Available: http://fossilworks.org. 
6. Gianechini FA, Apesteguía S, Landini W, Finotti F, Valieri RJ, Zandonai F. New abelisaurid remains from the Anacleto Formation (Upper Cretaceous), Patagonia, Argentina. Cretaceous Res. 2015; 54: 1-16.
7. Benyoucef M, Läng E, Cavin L, Mebarki K, Adaci M, Bensalah M. Overabundance of piscivorous dinosaurs (Theropoda: Spinosauridae) in the mid-Cretaceous of North Africa: The Algerian dilemma. Cretaceous Res. 2015; 55: 44-55.
8. Weishampel DB, Dodson P, Osmólska H. The Dinosauria. 2nd ed. Berkeley: University of California Press; 2004.
9. Carrano MT, Sampson SD. The Phylogeny of Ceratosauria (Dinosauria: Theropoda). J Syst Palaeontol. 2008; 6: 183-236. 
10. Allain R, Xaisanavong T, Richir P, Khentavong B. The first definitive Asian spinosaurid (Dinosauria: Theropoda) from the early cretaceous of Laos. Naturwissenschaften. 2012. doi: 10.1007/s00114-012-0911-7 
11. Carrano MD, Benson RBJ, Sampson SD. The phylogeny of Tetanurae (Dinosauria: Theropoda). J Syst Palaeontol. 2012; 10: 211-300. 
12. Tortosa T, Buffetaut E, Vialle N, Dutour Y, Turini E, Cheylan G. A new abelisaurid dinosaur from the Late Cretaceous of southern France: Palaeobiogeographical implications. Ann Paleontol. 2014; 100: 63-86.
