# Supplementary material for: The “χ” of the Matter: Testing the Relationship between Paleoenvironments and Three Theropod Clades
Source: PLoS One. 2016 Feb 1;11(2):e0147031. doi: 10.1371/journal.pone.0147031 (PMC4734717; doi:10.1371/journal.pone.0147031)
Supplement: S1 Letter — (PDF) [file pone.0147031.s004.pdf]

TERMS OF IMAGE USE AND RIGHTS OF ASSIGNMENT

I, Renata Floriano da Cunha, authorize the use of the image "The Sao Khua Formation Paleoenvironment", whose author is me, for the open-access journal PLoS ONE to publish under the Creative Commons Attribution License (CCAL) CC BY 4.0, as well as any further copyright asset of any products resulting from the hiring.

Renata F. Cunha.

____

Renata Floriano da Cunha

Curitiba, September 14th, 2015
