# Supplementary material for: The “χ” of the Matter: Testing the Relationship between Paleoenvironments and Three Theropod Clades
Source: PLoS One. 2016 Feb 1;11(2):e0147031. doi: 10.1371/journal.pone.0147031 (PMC4734717; doi:10.1371/journal.pone.0147031)
Supplement: S1 Table — (RTF) [file pone.0147031.s005.rtf]

Test	Remarks	
1	H0: There is no association between taxa and paleoenvironmental categories; the variables are independent.
H1: There is association between taxa and paleoenvironmetal categories; the variables are not independent.
Type of contingency table (lines x columns) for each analysis: 3x1
Statistical test applied: Chi-square test
Degree of freedom: 2	
2	H0: There is no association between taxa and paleoenvironmental categories; the variables are independent.
H1: There is association between taxa and paleoenvironmetal categories; the variables are not independent.
Type of contingency table for each analysis: 3x2
Statistical test applied: Chi-square test
Degree of freedom: 2	
3	H0: There is no association between taphonomic categories per taxa and paleoenvironmental categories; the variables are independent.
H1: There is association between taphonomic categories per taxa and paleoenvironmetal categories; the variables are not independent.
Type of contingency table for each analysis: 6x1
Statistical test applied: Chi-square test
Degree of freedom: 5	
4	H0: There is no association between taphonomic categories per taxa and paleoenvironmental categories; the variables are independent.
H1: There is association between taphonomic categories per taxa and paleoenvironmetal categories; the variables are not independent.
Type of contingency table for each analysis: 6x2
Statistical test applied: Chi-square test
Degree of freedom: 5	
5	H0: There is no association between abelisaurid taphonomic categories and paleoenvironmental categories; the variables are independent.
H1: There is association between abelisaurid taphonomic categories and paleoenvironmetal categories; the variables are not independent.
Type of contingency table for each analysis: 2x2
Statistical test applied: Chi-square test and Fisher's exact test
Degree of freedom: 1	
6	H0: There is no association between carcharodontosaurid taphonomic categories and paleoenvironmental categories; the variables are independent.
H1: There is association between carcharodontosaurid taphonomic categories and paleoenvironmetal categories; the variables are not independent.
Type of contingency table for each analysis: 2x2
Statistical test applied: Chi-square test and Fisher's exact test
Degree of freedom: 1	
7	H0: There is no association between spinosaurid taphonomic categories and paleoenvironmental categories; the variables are independent.
H1: There is association between spinosaurid taphonomic categories and paleoenvironmetal categories; the variables are not independent.
Type of contingency table for each analysis: 2x2
Statistical test applied: Chi-square test and Fisher's exact test
Degree of freedom: 1	
8	H0: There is no association between Cretaceous epochs and paleoenvironmental categories; the variables are independent.
H1: There is association between Cretaceous epochs and paleoenvironmetal categories; the variables are not independent.
Type of contingency table for each analysis: 2x2
Statistical test applied: Chi-square test and Fisher's exact test
Degree of freedom: 1	
